# The Arc of Migration and the Impact on Children’s Health and Well-Being: Forward to the Special Issue-Children on the Move

**DOI:** 10.3390/children6090100

**Published:** 2019-09-06

**Authors:** Charles Oberg

**Affiliations:** Division of Epidemiology and Community Health and Division of Global Pediatrics, University of Minnesota, Minneapolis, MN 55455, USA; oberg001@umn.edu

**Keywords:** children on the move, refugees, immigrants, trauma informed care, children’s rights

## Abstract

Since the start of this millennium the diaspora of families with children has continued unabated. UNICEF estimates that nearly 31 million children have been forcibly displaced from their homes by the end of 2017. This includes 13 million child refugees, an estimated 17 million children internally displaced within their own countries and over 900 thousand children seeking asylum in a different country. Even more staggering is the 75 percent increase in the number of child refugees between 2010 and 2015. This Special Issue, Children on the Move: The Health of Refugee, Immigrant and Displaced Children, examines in detail the health and well-being of our most vulnerable children today. It follows the arc of migration from home country, through transit and finally the challenges experienced in a child’s new country. The papers explore a variety of acute and chronic health conditions as well as the mental health of these children and youth. The articles examine the trauma experienced in their home country, the fleeing of war, violence and/or poverty, the travails during their journey, the stress throughout their stay in detention centers and refugee camps, and finally the transition to a new home country.

Since the start of this millennium the diaspora of families with children has continued unabated. The general population’s attention was drawn to the issue following the 2015 drowning of Alan Kurdi, the three-year-old Kurdish refugee from Syria. The photograph of the toddler lying face down on the shore made the journey palpable to millions worldwide. More recently, the image of children separated from their parents and held in detention centers on the southern border of the United States reawakened our collective awareness of the ongoing tragedy experienced by the global diaspora of families and their children.

UNICEF estimates that nearly 31 million children have been forcibly displaced from their homes by the end of 2017. This includes 13 million child refugees, an estimated 17 million children internally displaced within their own countries and over 900 thousand children seeking asylum in a different country [1]. Even more staggering is the 75 percent increase in the number of child refugees between 2010 and 2015 [2]. They are fleeing armed conflict, poverty, oppression and natural disasters, seeking to find a safe and welcoming new home. 

This Special Issue, *Children on the Move: The Health of Refugee, Immigrant and Displaced Children*, examines in detail the health and well-being of our most vulnerable children today. It follows the arc of migration from home country, through transit and finally the challenges experienced in a child’s new country. The articles examine the trauma experienced in their home country, the fleeing of war, violence and/or poverty, the travails during their journey, the stress throughout their stay in detention centers and refugee camps, and finally their transition to a new home country. It contains contributions from an international community of professionals working on behalf of all refugee, immigrant and displaced children. The papers explore a variety of acute and chronic health conditions as well as the mental health of these children and youth with an emphasis on trauma informed care. The contributions are grounded in the principles, standards and norms of child rights as articulated by the UN Convention on the Rights of the Child (CRC). All the articles provide new insights and strategies on how best to ground our clinical work as well as advocating for programmatic and policy change through a child rights based framework.

The Special Issue begins with an editorial by Stephanie Ettinger de Cuba, Allison Bovell-Ammon and Diana Becker Cutts that eloquently speaks to the “invisible walls” that are created through global policies within and across nations that first fail to address the antecedents of poverty, violence and climate change, and then isolationist and xenophobic polices that limit migration across borders. This is best exemplified by the present policy of the United States regarding its southern border and the especially harmful effect it is having on children and their families.

Nina Frield and Oliver Muensterer provide a glimpse into the hospitalization of refugee children admitted to hospitals in Germany. When families migrate from transitional refugee camps to hopefully their final destination, the morbidity and health burden frequently migrates with them. Their study is a retrospective review over a 10-year span. They found that hospitalized refugee children were significantly more likely to experience anemia, colonized with Methicillin-resistant *Staph aureus*, minor trauma, and esophageal foreign bodies as compared to non-refugee children. The results speak to the need to remain vigilant in the screening and assessment of refugee children once they have reached their destination.

Allison Bovell-Ammon, Stephanie Ettinger de Cuba, Sharon Coleman and colleagues provide an important paper from the Children’s Healthwatch collaborative that highlights a worrying trend of increased food insecurity rates and decreased participation in the Supplemental Nutrition Assistance Program (SNAP) amongst children born in the United States to immigrant families. This investigation of economic and nutritional hardship complements the other articles in the Special Issue and completes the arc regarding the family diaspora over the last decade. Specifically, the ongoing stress that accompanies the migration of families from their country of origin to the assimilation to a new home. The findings have major policy implication for the United States’ policies toward immigration and identifies that restrictive policies do have human consequences for children and youth.

Asterios Kampouras, Georgios Tzikos, Eustathios Partsanakis and colleagues examine the prevalence of disease and its resultant morbidity in the refugee camps on mainland Greece. A full 45% percent of those seen in the primary care office were infants, children and adolescents. The paper explores the magnitude of acute and chronic infectious diseases as well as non-communicable conditions including those associated with trauma and/or stress. The evolution in the approach to sheltering from a short-term way station into a long term encampment occurring during the winter months is poignant. It speaks to the adversity faced by these families as well as the need for policies by countries who are receiving migrating families with children.

The article by Andie Buccitelli and Myriam Denov explores the barriers experienced by young refugees fleeing war and conflict who are seeking asylum in Quebec, Canada. They face a myriad of care systems that are insensitive to their needs, values and beliefs. Trauma is a consistent and pervasive experience that spans from their home country, dangerous travels and resettlement at their destination. The paper examines a number of alternative approaches that were generated from the youths’ insights. These approaches validate their own identity and lived experiences so as to facilitate adjustment to their new home now as well as their lives for the foreseeable future.

Eileen Crespo highlights in her review the importance of oral health for newly arrived children in the United States. For these new arrivals, oral health is often a significant issue, with the severity of dental disease varying by the country of origin as well as cultural beliefs that can hinder access to care. It concludes with a call to health care providers to recognize oral health problems, make appropriate referrals and communicate effectively with families on the need for both preventive oral health and care.

Kathleen Miller, Calla Brown, Maura Schramko and Maria Svetaz provide a review of trauma-informed care approaches designed to address the toxic stress and trauma experience by refugee youth. It outlines 10 clinical pearls that provides guidance to providers who are caring for this vulnerable group of children and youth. These included utilizing a strength-based approach to care, creating an immigrant-friendly environment that promotes trusting relations, and utilizing a two-generational approach to care and advocacy both within the clinic and community.

Logan DeBord, Kali Hopkins and Padma Swamy provide a unique insight from the trainee perspective on providing care for children on the move. They provide two case studies that highlight the need for quality interpreters/translators when working with refugee and immigrant families. They also provide recommendations on the need for medical education changes designed to address this issue.

Ranit Mishori provides an introduction to the topic of age assessment of minors crossing international borders and the difficulty of establishing the correct assessment of the age of immigrant and refugee children. This introduction provides a summary of the best practices presently available in estimating a child’s chronological age. It discusses how medical knowledge can be misused during this sentinel event and concludes with a series of recommendations on adopting best policies, procedures and practices in the initial assessment and evaluation.

Ziba Vighri, Zoë Tessier and Christian Whalen**,** in recognition of the 30th anniversary of the UN Convention on the Rights of the Child (CRC), examine in depth the rights to provide for and protect children who have been displaced from their homes due to violence and war. The manuscript outlines the ramifications on children’s health and development. It provides an in-depth analysis of the mandated provisions of the CRC dedicated to protecting displaced and asylum-seeking children.

Melissa Diamond and Charles Oberg provide a brief report on the trauma experience of Syrian refugee families and their children presently living in Turkey. It explores gender related trauma such as sexual violence, domestic violence and cultural constructions of masculinity, as well as the impact on the educational experience of Syrian children. The paper concludes with a call for increased integration of parent trauma support in educational intervention trainings and the creation of safe spaces where mothers and fathers can discuss their own trauma and in hope of enhancing educational program efficacy for their children.

Avinash Shetty provides a comprehensive review of infectious diseases experienced by refugee children. The review focuses on the notable infectious diseases of active tuberculosis, HIV, hepatitis B and C and malaria as well as other parasitic infections. It also highlights vaccine preventable diseases. It provides a summary of health assessment guidelines for newly arriving immigrant and refugee children.

The Special Issue concludes with an opinion piece by the Special Issue Guest Editor, Charles Oberg on the *Budapest Declaration*, summarizing this landmark document on the rights of immigrant and refugee children [3]. It also discusses how as child health providers we must alter our approaches to a holistic plan based on a child rights based approach (CRBA) that melds the intersection between health care, health systems, policies and programs [4]. It demands we advocate for a new approach in the 21st century to global migration and the health and well-being of children and youth who are on the move.
